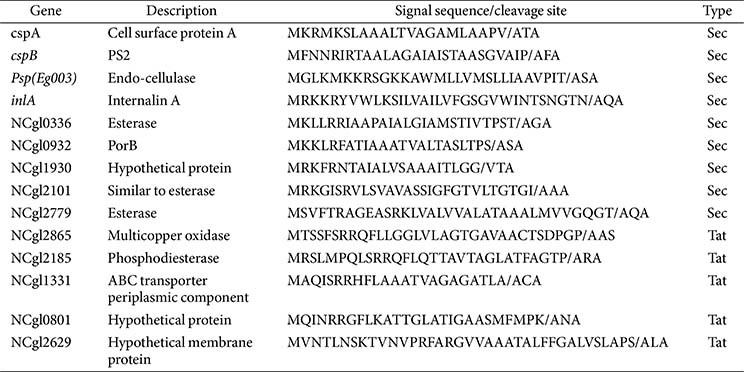# Secretory Expression of *Escherichia coli* L-Asparaginase in *Corynebacterium glutamicum*

**DOI:** 10.4014/jmb.2508.08051

**Published:** 2025-11-18

**Authors:** Ho-Seok Yoo, Jin-Young Lee, Su-Kyoung Yoo, Geun-Joong Kim

**Affiliations:** Department of Biological Sciences, and Institute of Sustainable Ecological Environment, College of Natural Sciences, Chonnam National University, Gwangju 61186, Republic of Korea

**Keywords:** L-asparaginase, secretory expression, signal sequence, *Corynebacterium*

## Abstract

L-Asparaginase is a potential therapeutic enzyme used in the treatment of acute lymphoblastic leukemia. It is also useful as a food processing aid. Hence, many studies have been conducted to develop and optimize production methods for L-asparaginase using various microbial hosts. In this study, a secretory expression route for L-asparaginase was developed using recombinant *Corynebacterium glutamicum*. Fourteen signal sequences were primarily mined and used to induce the secretion of *Escherichia coli* L-asparaginase II (AsnB) in *C. glutamicum*. The signal sequence ss2629 induced efficient secretion of AsnB, achieving a productivity of 25.4 mg/l in batch cultivation. The resulting Cg-AsnB in the culture supernatant was subsequently purified using anion exchange and size exclusion chromatography, resulting in an overall yield of >12.8 mg/l. Although the productivity and purification yield remained to be further improved, the overall biochemical and structural properties of purified Cg-AsnB were comparable to those of commercially available Ec-AsnB. Taken together, these results could provide an alternative platform for the secretory production of L-asparaginase using endotoxin-free *C. glutamicum* as a host.

## Introduction

L-asparaginase (L-asparagine amidohydrolase; EC3.5.1.1) catalyzes the conversion of L-asparagine to L-aspartic acid and ammonia. It is currently used for the treatment of acute lymphoblastic leukemia (ALL) and acute myeloid leukemia (AML) [1, 2]. It is also used as a processing aid in the food industry to prevent acrylamide formation [3]. Moreover, it has attracted considerable attention because of the physiological importance of its major (high-affinity) and minor (low-affinity) substrates, asparagine and glutamine, respectively, in triggering quorum sensing in various pathogens and enhancing metabolic function in cancer cells [4, 5]. Hence, basic studies on the structure-function relationships of these enzymes, and their high-level production for practical applications, have been steadily conducted at both academic and industrial levels. Various L-asparaginases from plants, animals, and microorganisms have been comprehensively studied for their biochemical properties and promising functions [6]. For example, L-asparaginase with low intrinsic L-glutaminase activity or high stability have been screened to reduce the side effects associated with high L-glutaminase activity during the treatment of ALL, thereby extending the potential scope for *in vivo* and *in vitro* applications [7, 8].

Given the potential of L-asparaginases, many studies have attempted to optimize their production under various conditions using different microbial hosts [6, 9]. In particular, attempts have been made to efficiently produce two types of L-asparaginases isolated from *Escherichia coli* and *Erwinia chrysanthemi* because of their relatively high activity and stability [9]. Moreover, given the inhibition effect of L-asparaginase from *E. coli* on solid cancer cells, it has the potential to serve as a novel drug for the treatment of these cancers [10, 11]. In recombinant *E. coli*, these L-asparaginases have generally been produced intracellularly [12], extracellularly [13], or in the periplasmic space [14] using combination of signal sequences and transport systems. They have been subsequently purified using fractionation, filtration, and chromatography to remove impurities, especially endotoxins. Although these production schemes have been able to achieve high yield and purity of L-asparaginases, alternative production strategies using *Bacillus subtilis* [15] and *Pichia pastoris* [16] as expression hosts have also been frequently explored [17]. This is because these strains are generally recognized as safe (GRAS) and are suitable for secretory expression. As endotoxin removal is not required, the purification process is relatively simple. For interesting yet unknown reasons, L-asparaginase produced by microorganisms, particularly *E. coli*, undergoes complex post-translational modifications (PTMs) [18]. These PTMs induce changes in protein properties. In some cases, they also cause side effects, such as acute immune response [19]. Therefore, further comparative studies on high-level secretory production and/or PTM patterns of L-asparaginase from alternative hosts, especially GRAS strains, are needed.

*Corynebacterium glutamicum* is a promising alternative host for the secretory expression of target proteins. As this strain is gram-positive and GRAS, it has been widely used for the production of valuable metabolites, such as amino acids and nucleic acids [20]. It is also well known that this strain secrets only a limited number of proteins into the culture medium, therefore, its detectable extracellular proteolytic activity is extremely low [21, 22]. Considering these properties, the functional secretion of the protein of interest (POI) from *C. glutamicum* may be advantageous in terms of simpler purification and better production yields for practical applications.

In this study, we aimed to develop a novel system for producing *E. coli* L-asparaginase II (AsnB) through secretory expression in *C. glutamicum* using a screened signal sequence. To this end, we systematically mined suitable signal sequences dependent on the Tat or Sec system and compared their secretory expression abilities against *C. glutamicum*-expressed AsnB (Cg-AsnB). We assessed the secretion profile and yield of the screened clone expressing Cg-AsnB containing the finally selected NCgl2629 signal sequence (ss2629) through batch cultivation. Moreover, we compared the structural and functional properties of purified Cg-AsnB with those of the commercially available asparaginase Ec-AsnB.

## Materials and Methods

### Bacterial Strains, Recombinant Plasmids, and Culture Conditions

*E. coli* XL1-Blue was used as a host for gene cloning and DNA manipulation. *C. glutamicum* (ATCC 13032) was used as an expression host for the recombinant L-asparaginase Cg-AsnB. The corresponding gene encoding AsnB (GenBank Accession No. P00805) without the innate signal sequence was amplified by polymerase chain reaction (PCR) using the chromosomal DNA of *E. coli* K-12 as a template and introduced into the shuttle vector pXMJ19 [23] using the restriction sites *Hin*dIII and *Eco*RI. To introduce the mined signal sequences into the resulting construct, the recognition site of *Hin*dIII was replaced with the *Eco*RV site by circular PCR using a primer set. The PCR-amplified signal sequences (ssCspA, ssCspB, ssPsp, ssInlA, ss0336, ss0932, ss1930, ss2101, ss2779, ss2865, ss2185, ss1331, ss0801, and ss2629) were then inserted using the *Eco*RV restriction site (Fig. S1). All strains and plasmids used in this study are listed in Table 1, and the sequences of the signal sequences used in this study are listed in Table 2.

Recombinant *C. glutamicum* was routinely cultured in 20 ml of Luria-Bertani (LB) medium (10 g/l tryptone, 5 g/l yeast extract, and 10 g/l NaCl) supplemented with chloramphenicol (10 μg/ml) in a 100 ml flask using a rotary shaker (200 rpm) at 30°C. The growth of recombinant cells was monitored by measuring the optical density at 600 nm (OD_600_) using a spectrophotometer (UV-1601, Shimadzu, Japan). Protein expression was induced with 0.5 mM isopropyl-β-D-1-thiogalactopyranoside (IPTG) when the OD_600_ was approximately 0.8–0.9.

### Analysis of Secretory Expression

To analyze the secretory expression of Cg-AsnB using mined signal sequences, each cultured cell was harvested by centrifugation (13,000 ×*g*, 20 min), resuspended in 50 mM Tris–HCl buffer (pH 8.6), and lysed by sonication (25% amplification, 3 min). The resulting sample was used as a control for intracellular expression of the recombinant protein. The secreted protein in the culture medium was recovered by precipitation with cold acetone. In brief, 0.3 ml of the culture supernatant was mixed with three volumes of cold acetone and incubated overnight (18 h) at –20°C. The precipitate was then collected by centrifugation at 13,000 ×*g* for 30 min at 4°C, washed once with cold acetone, and centrifuged again under the same conditions. The resulting pellet was resuspended in Tris–HCl buffer (pH 8.6). All protein samples were quantified using the Bradford assay and analyzed by electrophoresis using a 15% SDS–polyacrylamide gel. Subsequently, the gel was stained with Coomassie Brilliant Blue dye, and additional densitometry analysis was performed if necessary.

### Quantitative Analysis of L-Asparaginase Activity

L-Asparaginase activity was assessed by measuring the absorbance at 436 nm using 176 mM L-asparagine as the substrate in 50 mM Tris–HCl buffer (pH 8.6) with Nessler reagent (Sigma-Aldrich, USA), as described by Shifrin *et al*. [24]. One unit (U) of L-asparaginase activity was defined as the amount of enzyme required to release 1 μmol of ammonia per minute at 37°C.

### Laboratory-Scale Production of Cg-AsnB Using High-Cell-Density Batch Culture

Laboratory-scale production of Cg-AsnB from recombinant *C. glutamicum* was performed using a 5 L jar-bioreactor (KoBioTech, Republic of Korea) with a working volume of 2 L. A seed culture (1%, v/v) was inoculated into the defined medium [40 g glucose, 10 g (NH_4_)_2_SO_4_, 1 g KH_2_PO_4_, 1 g K_2_HPO_4_, 3 g yeast extract, 0.5 g MgSO_4_·7H_2_O, 10 mg FeSO_4_·7H_2_O, 10 mg MnCl_2_·4H_2_O, 10 mg ZnSO_4_·7H_2_O, 10 mg CaCl_2_·2H_2_O, 1 mg CuCl_2_·4H_2_O, 0.1 mg biotin, and 1 mg thiamin per liter] supplemented with chloramphenicol (10 mg/l). The resulting cells were cultured at pH 7.0 and 32°C, with the agitation speed ranging from 200 to 800 rpm to maintain appropriate O_2_ saturation. The pH of the culture medium was maintained using ammonia solution. The air flow rate was maintained at 1.0 vvm, and foam was controlled using sterilized Antifoam B Emulsion (Sigma-Aldrich). During the culture, the amount and activity of secreted Cg-AsnB were analyzed at the indicated times using the sampled culture medium.

### Purification of Cg-AsnB

After 14 h of high-cell-density batch culture, a sample of the culture (100 ml) was used for protein purification. Cells were removed by centrifugation at 13,000 ×*g* for 20 min. The resulting supernatant containing secreted Cg-AsnB was filtered through a 0.2 μm syringe filter, diluted with four volumes of binding buffer (20 mM Tris–HCl, pH 8.6), and loaded onto a Resource HiTrap Q HP anion exchange column (Cytiva Life Sciences, USA) pre-equilibrated with binding buffer. After thorough washing with the same binding buffer, bound proteins were eluted using a linear gradient with five column volumes of binding buffer containing 0–1 M NaCl. The eluted fraction containing Cg-AsnB was further purified by size exclusion chromatography (SEC) in a Superdex 200 Increase 10/300 GL column (Cytiva Life Sciences) using 20 mM Tris–HCl buffer (pH 7.3) containing 150 mM NaCl, as required. After purification, the purity and yield of the purified Cg-AsnB were determined using 15%SDS–PAGE and densitometry (GS700, Bio-Rad, USA). Protein concentration was measured with the Bradford method using bovine serum albumin (BSA) as a standard.

### Structural Properties of Recombinant Cg-AsnB

The quaternary structure of recombinant Cg-AsnB was analyzed by SEC using a Superdex 200 Increase 10/300 GL column mounted on an FPLC system (AKTA, Cytiva Life Sciences), according to the manufacturer’s instructions. A calibration curve was constructed by plotting the elution volume (Ve)/void volume (Vo) ratio against the logarithm of the molecular weights (MWs) of protein size markers (carbonic anhydrase, ovalbumin, conalbumin, and aldolase); this was used to determine the MW of Cg-AsnB (150 μg) at a constant flow rate 0.5 ml/min [25]. The intrinsic fluorescence of purified Cg-AsnB (0.7 μM) was recorded using a spectrofluorometer (Infinite-200, Tecan Group Ltd., Switzerland) under the defined conditions (at 25°C using Tris–HCl, pH 8.6) at an excitation wavelength of 280 nm and an emission wavelength of 250–700 nm.

### Two-Dimensional Gel Electrophoresis (2-DE)

The PTM patterns of Cg-AsnB expressed in *C. glutamicum* were analyzed using 2-DE (ProteomeTech Inc., Republic of Korea). For a clearer comparison, the commercially available asparaginase Ec-AsnB (MEDAC, Germany) was used as a control. The proteins were initially separated by isoelectric focusing using immobilized nonlinear gradient strips (pH 3.0–10.0). Second– dimensional separation was then performed on a 10%–16%gradient SDS-polyacrylamide gel. After 2-DE separation, proteins were detected by staining with Coomassie brilliant blue.

### Assessment of Protein Stability and Effects of Additives on Enzyme Activity

To determine storage stability, purified Cg-AsnB was stored at 4°C for 21 days. Samples were collected at various time points to measure residual activity [26]. Ec-AsnB was used as a control for a clearer comparison under the same conditions. Resistance to proteinase K digestion was assessed at 37°C for 0–45 min using solutions containing purified Cg-AsnB (5 μg) and proteinase K (0.01 mU). After incubation for the indicated time, each reaction solution was mixed with the sample buffer and analyzed on a 15% SDS-polyacrylamide gel [27]. The effects of various additives on enzyme activity were also assessed, as reported previously [28, 29].

## Results

### Mining of Signal Sequences for Secretory Expression of Cg-AsnB in *C. glutamicum*

To secrete a heterologous protein from a surrogate host, identifying appropriate signal sequences and constructing the correct fusion with the POI are crucial steps for practical applications. However, as the secretion efficiency of a protein is affected by the combination of the signal sequence and cargo protein, there is no general method for selecting a signal sequence for a specific cargo protein. Therefore, to identify signal sequences suitable for the secretion of recombinant Cg-AsnB, 14 candidate signal sequences (Table 2) derived from *C. glutamicum* and other strains (*C. ammoniagenes*, *Paenibacillus* sp., and *Listeria monocytogenes*) were arbitrarily selected based on previously reported functional secretion studies and extracellular proteome analyses [30, 31]. This mining process considered the size of the secreted cargo protein, the amount of secreted protein, and the differences in the sequences compared with previously known signal sequences. Additionally, the putative signal sequences and cleavage sites were analyzed using SignalP software, followed by prediction of secretion pathways. The mined signal sequences were predicted to be Sec-dependent (nine types) and Tat-dependent (five types) sequences.

**Fig. 1:**
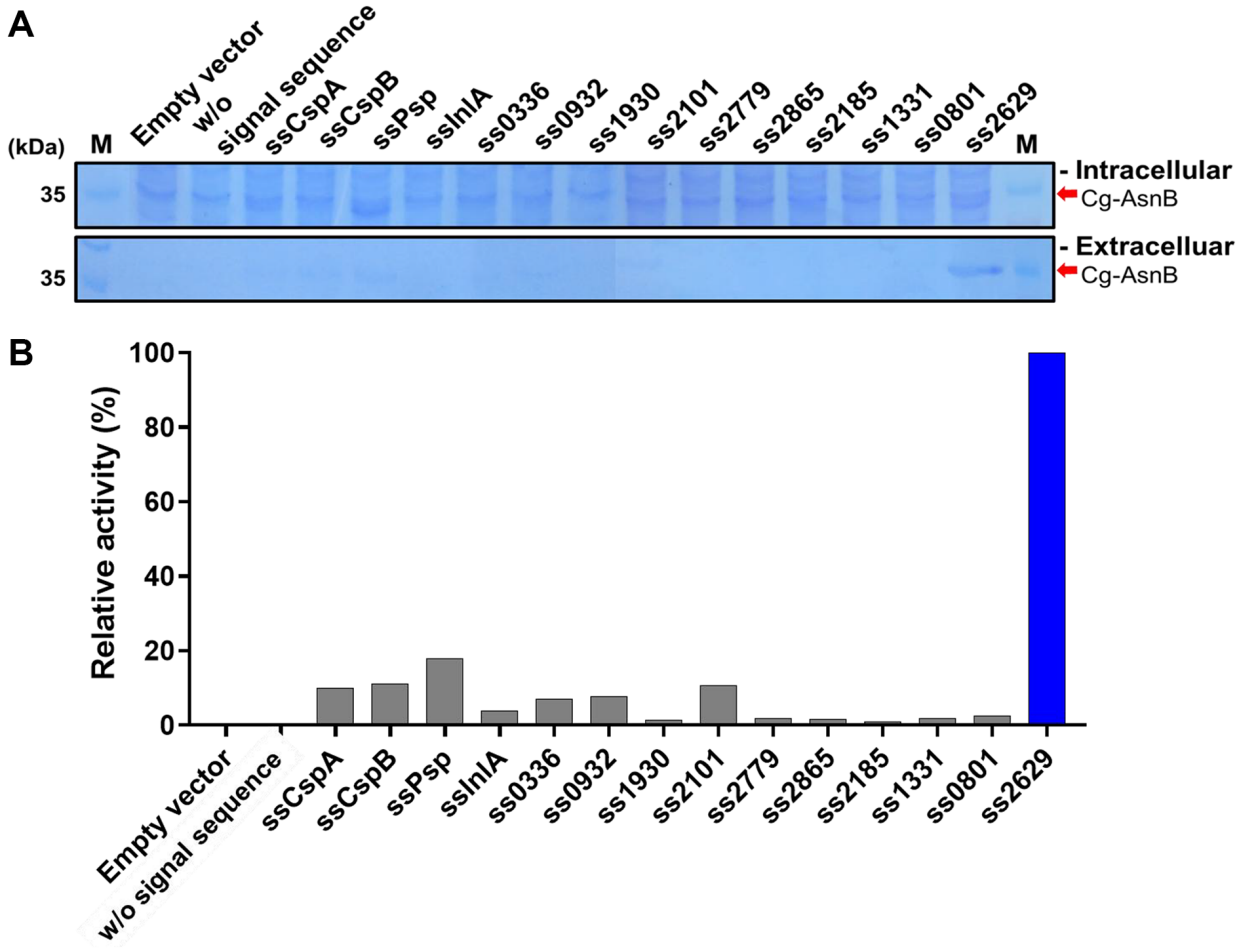
Comparison of the secretion ability of mined signal sequences using *Corynebacterium glutamicum* as a host. (**A**) SDS-PAGE analysis of the recovered cells and supernatant to compare the total expression and secretion levels of Cg-AsnB. Cells and supernatant corresponding to 75 μl of culture were analyzed under the same conditions. (**B**) Comparison of Cg-AsnB activity detected in the culture supernatant in panel (**A**). Relative activity was calculated by normalizing the clone with the highest activity to 100%. Lane M, molecular weight marker.

To determine whether the mined signal sequences could induce the functional secretion of Cg-AsnB, each signal sequence was incorporated into the N-terminus of AsnB lacking the innate signal sequence using the pXMJ19 shuttle vector containing the IPTG-inducible *tac* promoter (Fig. S1). Accurate integration of each signal sequence was confirmed by DNA sequencing, as each DNA fragment encoding the signal sequence was fused by blunt-end ligation using the *Eco*RV restriction site. After confirming accurate orientation and cloning, all recombinant cells were cultured in LB medium under the conditions described in the Materials and Methods section. The secretory expression and resulting activity of Cg-AsnB were then analyzed. As shown in Fig. 1, recombinant Cg-AsnB fused with seven signal sequences (ssCspA, ssCspB, ssPsp, ss0336, ss0932, ss2101, and ss2629) exhibited reproducibly detectable activity in the culture medium. However, a distinct band corresponding to the expected size of Cg-AsnB appeared only in clones containing the signal sequence ss2629. These results suggested that Cg-AsnB was effectively secreted in a Tat-dependent manner in the heterologous host *C. glutamicum*. Hence, the clone harboring the signal sequence ss2629 was used for further analysis.

### Secretory Production of Cg-AsnB in Flask and Laboratory-Scale Batch Cultures

**Fig. 2:**
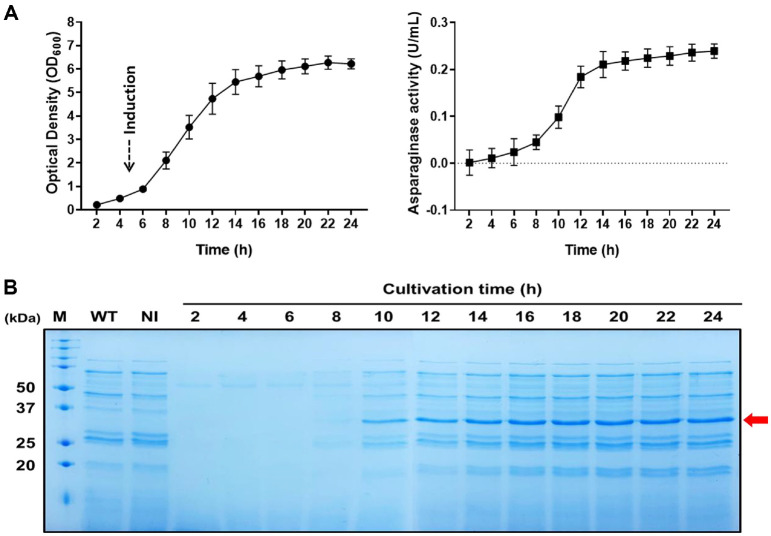
Secretory expression analysis of Cg-AsnB containing the signal sequence ss2629 in flask cultures. (**A**) Cell growth (●) and extracellular Cg-AsnB activity (■) profiles in flask cultures. (**B**) SDS–PAGE analysis of the culture supernatant after removing cells by centrifugation. At each time point, 300 μl of the culture supernatant was used for SDS– PAGE analysis. Lane M, molecular weight marker (kDa); WT, *C. glutamicum* ATCC 13032; NI, no IPTG induction; and 4-15, supernatant samples taken at the indicated culture time intervals. The red arrow indicates Cg-AsnB (34.6 kDa).

To further assess the secretory expression profile of Cg-AsnB containing the signal sequence ss2629, flask cultures (200 ml working volume in a 1 L flask) were performed under the aforementioned conditions for 24 h, and the expression level and activity of Cg-AsnB were analyzed. As shown in Fig. 2A, the activity of Cg-AsnB was distinctly detected in the culture medium after 2 h of induction with IPTG. This activity gradually increased over time. SDS–PAGE analysis revealed that the expression level of Cg-AsnB in the culture supernatant also increased proportionally, reaching a plateau after 8 h of induction (Fig. 2B). No significant growth retardation was observed in cells expressing heterologous recombinant Cg-AsnB compared with those carrying the empty vector.

To determine whether the productivity of recombinant Cg-AsnB could be maintained in a relatively high-cell density culture, a batch culture of recombinant *C. glutamicum* expressing Cg-AsnB was performed in a 5 L jar fermenter. Recombinant cells were cultured in a well-known defined medium until glucose (40 g/l), initially supplied as the sole carbon source, was completely depleted. The supplied glucose was completely consumed after 14 h of culture, and the OD_600_ reached approximately 70 (Fig. S2). After removing the cells by centrifugation, the unconcentrated culture supernatant was analyzed by SDS–PAGE. After 4 h of induction, a band corresponding to Cg-AsnB was clearly observed and gradually increased as the absorbance of the cells increased. Based on the corresponding bands observed in SDS–PAGE and the measured enzyme activity, the amount of Cg-AsnB in the culture supernatant obtained from the 14 h batch culture was calculated to be approximately 25.4 mg/l. Contrary to expectations, this relatively low expression level is likely attributed to limitations in the inducer and culture time. Therefore, optimizing high-cell density cultures in terms of the culture time, and supplied amount of inducer and glucose is expected to significantly improve productivity.

### Purification and Characterization of Cg-AsnB

Recombinant Cg-AsnB was purified from the batch culture supernatant (100 ml) by sequential anion exchange and SEC. Approximately 1.3 mg of L-asparaginase was purified with high purity (>93%). The recovery yield was approximately 52%. The specific activity of purified Cg-AsnB was determined to be 98 ± 6 U/mg protein, which is 110%–115% of that of the commercially available L-asparaginase Ec-AsnB under the same conditions. Moreover, limited L-glutaminase activity (2%–4%) relative to L-asparaginase activity was detected in recombinant Cg-AsnB under the same assay conditions described in a previous report [32].

**Fig. 3:**
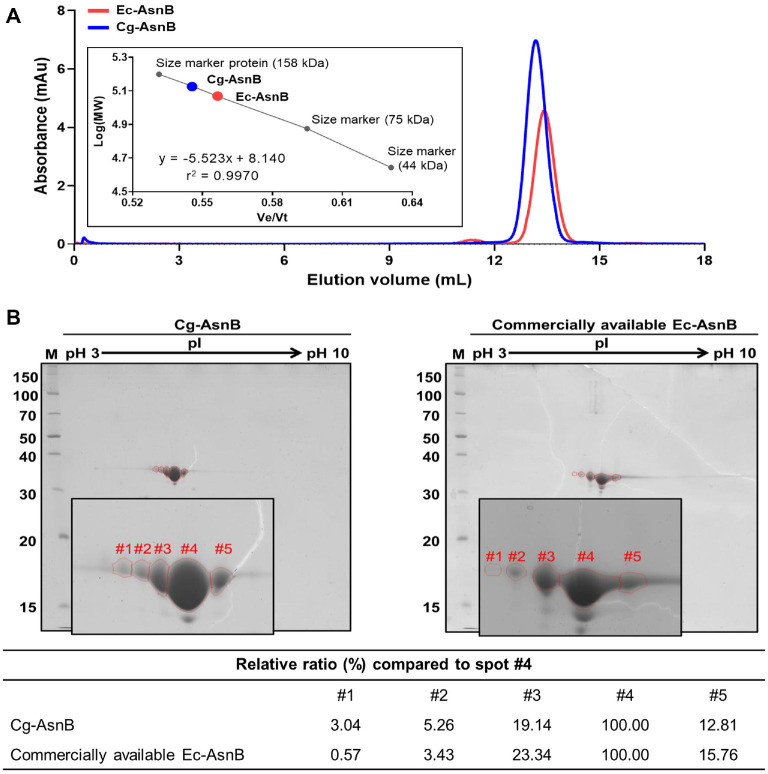
Comparison of structural properties between Cg-AsnB and Ec-AsnB. (**A**) Elution profiles of individually loaded proteins using gel permeation chromatography. Purified Cg-AsnB (150 μg) was used to compare the elution profiles of the control and molecular weight marker proteins. The molecular mass of Cg-AsnB was calculated using a Ve/Vt-Log(MW) plot based on the relative elution profile of the marker protein (insert box). (**B**) Comparison of two-dimensional gel electrophoresis (2-DE) patterns of Cg-AsnB produced in this study (left) and those of commercially available Ec-AsnB (right). Using the same amount of protein (7.5μg), IPG strips (pH 3.0–10.0) were used for the first dimension and a 10%–16% gradient SDS-polyacrylamide gel was used for the second dimension. Numbered circles represent spots separated from each protein. Their relative ratios are calculated in the table below.

The apparent structural properties of Cg-AsnB were analyzed and compared with those of Ec-AsnB, focusing on quaternary structure, intrinsic fluorescence, and PTMs. The quaternary structure of Cg-AsnB was analyzed using SEC. As shown in Fig. 3A, Cg-AsnB and Ec-AsnB were eluted at 13.2 and 13.4 ml, respectively, and their molecular masses were calculated to be 126.6 and 113.8 kDa, respectively. Therefore, both these enzymes were determined to form a tetrameric structure, as reported previously [32, 33]. As an additional structural feature, the intrinsic fluorescence of Cg-AsnB was analyzed by scanning the emission spectrum at an excitation wavelength of 280 nm. Consequently, the fluorescence emission spectrum of Cg-AsnB was confirmed to be identical to that of Ec-AsnB with a λmax at 320 nm, suggesting structural similarity between the two enzymes (Fig. S3). This is consistent with spectrum reported in a previous study [14].

**Fig. 4:**
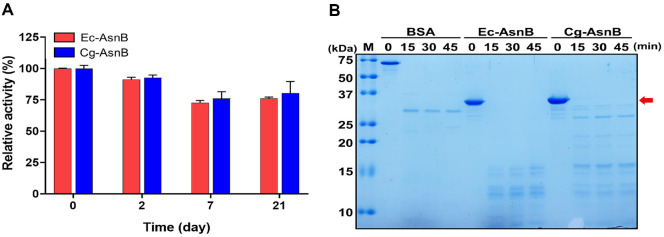
Storage stability and proteinase K susceptibility of recombinant Cg-AsnB. (**A**) Analysis of the remaining activity of purified Cg-AsnB during storage at 4°C for 21 days. Ec-AsnB was used as a control under the same conditions. (**B**) Comparison of protease susceptibility of Cg-AsnB and Ec-AsnB. Equal amounts of each protein (5 μg) were treated with protease K (0.01 mU) at 37°C for the indicated times.

As an unexpected but interesting structural feature, recombinant L-asparaginases have been reported to possess a complex pattern of modifications, including phosphorylation [18]. Hence, we performed 2-DE to determine whether the known modifications of L-asparaginase were also present in the gram-positive host *Corynebacterium* and compared them with those of Ec-AsnB. Both Cg-AsnB and Ec-AsnB showed similarly resolved patterns in 2-DE (Fig. 3B), despite being expressed in different hosts. Although these results indicate no significant structural differences between the two enzymes, we speculated that the slight difference in relative concentration between the resolved spots of the two enzymes on 2-DE could influence the specific activity. These differences in relative concentration between 2-DE spots could also influence the stability of the enzymes. To verify this assumption, we compared the stability of recombinant Cg-AsnB from *Corynebacterium* with that of Ec-AsnB as a control. As shown in Fig. 4A, no significant difference was noted in the stability of the two enzymes when stored at 4°C for 21 days. On assessing the resistance to proteinase K treatment, the digestion patterns between the two enzymes were indistinguishable when treated with the same concentrations of protease K (Fig. 4B). Taken together, these results strongly suggest that the stability of recombinant Cg-AsnB from *Corynebacterium* is at least comparable to that of commercially available Ec-AsnB.

We also conducted experiments to determine whether the enzyme activity changed on adding various additives, including divalent metal ions [34, 35]. The activity of both Cg-AsnB and Ec-AsnB was commonly reduced when metal ions (2 mM) were added. Interestingly, the activity increased by more than 50% when DTT, a reducing agent, was added to the reaction mixture (Fig. S4). Other general characteristics, such as optimum pH (7.0–8.0) and temperature (40°C–45°C), were also similar to those mentioned in the original report characterizing the wild-type *E. coli* L-asparaginase AsnB [36].

## Discussion

Secretion strategies for heterologous protein expression have many advantages for the production of the POI; this can facilitate downstream purification procedures and avoid the contamination of cellular components, especially when using gram-positive strains as hosts [37]. *C. glutamicum*, a promising gram-positive host for protein secretion, has two major secretion pathways, namely Sec- and Tat-dependent pathways, which function in different ways depending on the signal sequences and folding state of cargo proteins [38]. So far, no distinct structural features can definitively determine which protein is better suited to one of these two systems. Nonetheless, studies on the secretion of heterologous proteins using specific signal sequences of *C. glutamicum* have been successfully conducted [39-41].

In this study, *C. glutamicum* was used as a host for the secretory expression of AsnB. Comparative analysis using 14 signal sequences revealed that recombinant Cg-AsnB was effectively secreted in a Tat-dependent manner when fused with the signal sequence ss2629. We further assessed the universality of this signal sequence for the secretion of heterologous proteins using two reporter proteins (GFPuv and Rluc) as model cases. However, when this signal sequence was fused to each of the two proteins, the secretion efficiency was much lower than expected. The highest secretion efficiency was observed with ss0801 (Fig. S5), despite its low efficiency in secreting Cg-AsnB. Interestingly, the ss0801 signal sequence also functions in a Tat-dependent manner. This reconfirms the well-known fact that it is very difficult to identify an efficient secretion signal sequence that can be commonly applied to various proteins. Thus, the process of screening the most suitable signal sequence for each POI is crucial.

As demonstrated in this study, the secretory production of Cg-AsnB using the signal sequence ss2629 has practical applicability. In flask cultures, Cg-AsnB was secreted at levels accounting for up to 18%–23% of the total extracellular protein. In addition to these promising results, no significant expression of stress response proteins (chaperones and proteases) or growth retardation was observed. This trend was also observed in laboratory-scale batch cultures; however, the secretion levels were lower than those noted in flask cultures in terms of productivity. This is because the increase in productivity was limited compared to the increased amount of cells. These problems may arise from shear stresses due to excessive agitation with the rapid increase in cell mass, the stability of secreted proteins, the effect of surfactants treated as antifoaming agents, and the proteolytic enzymes released from lysed cells. A more fundamental problem is that differences in media composition between flask (LB) and laboratory-scale (defined media containing yeast extract) cultures may be closely related to expression levels.

In addition to these factors, the main reason for the relatively low productivity was the limited amount of inducer supplied. Because the inducer was only added at a final concentration of 0.5 mM, sufficient protein expression was not induced as the cell density increased until the final OD_600_ was 70. We had conducted a batch culture experiment by additionally supplying the inducer under varying conditions. When a different amount of glucose (50 g/l) was initially supplied as the sole carbon source and the inducer (IPTG) was proportionally added up to concentrations exceeding 2.5 mM in response to increasing cell mass, the OD_600_ reached approximately 100. Moreover, the amount of secreted Cg-AsnB was measured to be approximately 300–350 mg/l (Fig. S6). We are currently further conducting experiments to confirm the reproducibility of these results and to further improve the secretion efficiency of Cg-AsnB using high cell-density fed-batch cultures.

Interestingly, PTMs of AsnB have been reported in a previous study; however, the exact mechanisms underlying these modifications, including technical derivatives, and their impact on structure and function, including their adverse effects as a drug, remain unclear [18]. As shown in Fig. 3B, these modifications are also commonly observed in other expression systems using a gram-positive strain as a host; however, the relative proportions of spots according to modifications on 2D gels may slightly vary between host strains. Therefore, it would be interesting to explore the origin (biological or technical) of modifications and the functional roles of these PTMs *in vivo*.

In conclusion, this study paves the way for the secretory production of Cg-AsnB by a Tat-dependent signal peptide using the endotoxin-free gram-positive strain *C. glutamicum*. Recombinant Cg-AsnB was effectively secreted in flask cultures and laboratory-scale batch cultures, and was easily purified by successive steps of column chromatography. The structural and functional properties of the resulting purified Cg-AsnB were comparable to those of commercially available Ec-AsnB. Although further process optimization in terms of secretion level and production yield is still required, this study provides a basis for the practical production of Cg-AsnB from *C. glutamicum*. Mined signal sequences may also be useful for the extracellular secretion of a heterologous POI.

## Figures and Tables

**Table 1:** Strains and plasmids used in this study.

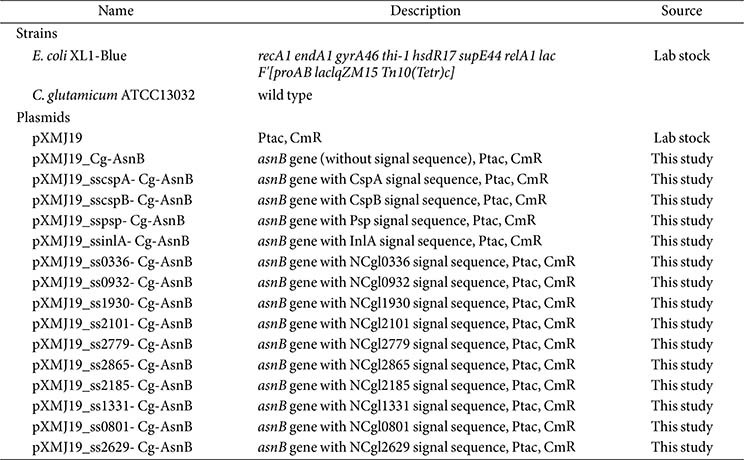

**Table 2:** Signal sequences used for Cg-AsnB secretion in this study.